# Endocannabinoid System in Health and Disease: Current Situation and Future Perspectives

**DOI:** 10.3390/ijms21103549

**Published:** 2020-05-18

**Authors:** Rosaria Meccariello

**Affiliations:** Department of Movement Sciences and Wellbeing, University of Naples “Parthenope”, Via Medina 40, 80133 Naples, Italy; rosaria.meccariello@uniparthenope.it; Tel.: +39-081-5474668

The endocannabinoid system (ECS) is a complex signaling system that includes cannabinoid receptors, their endogenous ligands (endocannabinoids), and biosynthetic and hydrolytic enzymes [1]. ECS controls many basic biological processes, and exerts its activity in the nervous system and in peripheral tissues, with direct involvement in synaptic plasticity and neuroprotection, pain control, mood and behavior, learning and memory, stress response, reproduction, fertility and pregnancy, food intake and energy balance, immune response, cancer progression, and much more [2,3,4,5,6,7,8,9,10,11,12,13,14]. As a consequence, ECS impairment has been reported in several diseases, and its modulation currently represents a possible therapeutic strategy for the treatment of neurodegenerative, reproductive and inflammatory diseases, obesity and metabolic syndrome, or cancer, among many others. Hence the need to fully elucidate the molecular and epigenetic mechanisms related to ECS activity.

This Special Issue of the International Journal of Molecular Sciences is a collection of eight review articles and five original research articles, and offers an updated overview of current knowledge on ECS in both physiological and pathological conditions. 

An update on the epigenetics of the ECS has been reported in the review article by the group of Pierantoni, with focus on its emerging role in reproduction and fertility. ECS undergoes epigenetic modulation by life style and environmental factors, with consequences on health and disease. Nevertheless, the modulation of ECS induces epigenetic changes, to different extents, of target genes, with possible trans-generational effects in the off spring through the transmission of deregulated epigenetic marks in the gametes [15].

The activity of cannabinoid receptors, CB1 and CB2, in male germ cell development—from gonocyte up to mature spermatozoa—has been reviewed by Barchi et al.; interestingly, new roles in paternal epigenetic trans-generational inheritance and testis cancer are also discussed [16]. Furthermore, Chioccarelli et al. reveal a new role for CB1 in the mechanism of sperm chromatin condensation in the epididymis, a critical process for the extent of nuclear condensation of mature spermatozoa. Using *Cnr*1^-/-^ knockout mice, the authors demonstrate the requirement of CB1 activity, in driving the formation of inter-/intra-protamine disulphide bridges, and in histone removal in a mechanism involving the hyper-acetylation of histone H4, once again confirming the need of CB1 activity for the production of high-quality spermatozoa [17].

Moving toward female reproduction, Cecconi and coworkers report the expression of major endocannabinoid-binding receptors (CB1, CB2, GPR55 and TRPV1) at different stages of mouse oocyte maturation, and investigate the effects of specific receptor antagonists, revealing a new possible role for GPR55 and potentially offering new targets for the therapy of female reproductive alterations [18]. 

The involvement of CB1 in the hypothalamic control of food intake and reproduction has been investigated by Zuccarini et al., through the pharmacological and genetic manipulation of CB1 in zebrafish. The CB1-dependent effect on the guidance, fasciculation and routing of GnRH3 (Gonadotropin Releasing Hormone 3) and AgRP1 (Agouti-related peptide 1) axons has been demonstrated, suggesting the need of CB1 signaling in the correct development of neuroendocrine function related to food intake and reproduction [19]. 

In this respect, the gut–brainaxis represents a key regulator of the hypothalamic neuronal networks related to food-sensing and appetite. Therefore, the review article by Forte et al. summarizes the emerging role of an "expanded ECS", or endocannabinoidome (eCBome), encompassing endocannabinoid-like mediators in the modulation of the communication between microbiota and gut–brainaxis, with consequences on host metabolism and a critical role in obesity onset [20].

ECS may be a therapeutic target for the treatment of disease, and the modulation of endocannabinoid tone by the fatty acid hydrolyzing enzyme (FAAH) is a key step in ECS signaling. In this respect, the research article by Giacovazzo et al. compares different administration routes (i.e., intranasal, intraperitoneal and oral) to in vivo inhibit FAAH activity in mouse brains. Interestingly, the authors demonstrate the efficacy of intranasal FAAH inhibition, and suggest this delivery model as a suitable alternative to enhance the tone of endocannabinoids within the brain, for the treatment of neurodegenerative disorders and the improvement patients’ compliance [21].

The functional participation of the ECS in neurodegenerative disorders has been analyzed by Bhatia-Dey and Heinbockel, who focused a review article on the endocannabinoid-mediated neuromodulation in the olphactory bulb, a brain region in which neurogenesis continues throughout the life span, suggesting possible therapeutic interventions in decelerating neurodegenerative pathology [22]. 

In spite of the known involvement of CB2 in the neuorogenesis in both neuroinflammatory conditions and in response to pathophysiological stimuli, Mensching et al. demonstrate that stable adult neurogenesis occurs in the hyppocampus of *Cnr2^-/-^* mice, suggesting different signaling mechanisms for basal and damage-induced neurogenesis [23].

The Interplay between the ECS, epilepsy and cannabinoids has been reviewed by the group of Mitchell. The deep involvement of ECS in neuroinflammation points out the possible use of cannabinoids-based treatments to modulate ECS, as alternatives to more conventional pharmacological therapies, but also underlines the need for further research in the field to sustain the use of therapeutic cannabis [24].

The review article by Argenziano et al. analyzes the role of ECS in the pathogenesis and onset of pediatric inflammatory and immune diseases, like immune thrombocytopenia, juvenile idiopathic arthritis, inflammatory bowel and celiac disease, obesity and fatty liver disease, neuroinflammatory diseases, and type 1 diabetes mellitus [25].

As reviewed by Laezza et al., ECS can exert anticancer activity, modulating several signaling pathways involved in cell proliferation, differentiation, migration and angiogenesis [26], but their use in chemotherapeutic protocols requires further investigation, and is currently limited to the treatment of pain or chemotherapy-induced symptoms. However, in recent years, attention has been addressed to cannabidiol CBD, a non-psychotomimetic phytocannabinoid derived from the marijuana plant, *Cannabis sativa*, of particular interest for clinical application. In this respect, the review article by Kis et al. summarizes the anticancer effects of CBD in vitro and in vivo, and its main pharmacological and toxicological aspects [27]. 

Taken together, this Special Issue provides a very updated overview on ECS activity and modulation in physiological and pathological conditions, through the presentation and integration of data from experimental animal models and clinical observation. The deep involvement of ECS in the control of biological functions is far from fully clarified, but all the submitted manuscripts expand the knowledge in the field of ECS involvement in both health and disease, with a particular attention to the possible therapeutic exploitation. 

Lastly, the Editor hopes this Special Issue of the International Journal of Molecular Sciences may be useful for basic and clinical scientists working in the field, and deeply thanks all the authors who contributed with excellent works, and the reviewers engaged for the peer-review process.
